# NOS Inhibition Enhances Myogenic Tone by Increasing Rho-Kinase Mediated Ca^2+^ Sensitivity in the Male but Not the Female Gerbil Spiral Modiolar Artery

**DOI:** 10.1371/journal.pone.0053655

**Published:** 2013-01-03

**Authors:** Katrin Reimann, Gayathri Krishnamoorthy, Philine Wangemann

**Affiliations:** 1 Department of Anatomy and Physiology, Kansas State University, Manhattan, Kansas, United States of America; 2 Universitätsklinik für Hals-, Nasen- und Ohrenheilkunde, Eberhard-Karls Universität, Tübingen, Germany; Center for Cancer Research, National Cancer Institute, United States of America

## Abstract

Cochlear blood flow regulation is important to prevent hearing loss caused by ischemia and oxidative stress. Cochlear blood supply is provided by the spiral modiolar artery (SMA). The myogenic tone of the SMA is enhanced by the nitric oxide synthase (NOS) blocker L-N^G^-Nitro-Arginine (LNNA) in males, but not in females. Here, we investigated whether this gender difference is based on differences in the cytosolic Ca^2+^ concentration and/or the Ca^2+^ sensitivity of the myofilaments. Vascular diameter, myogenic tone, cytosolic Ca^2+^, and Ca^2+^ sensitivity were evaluated in pressurized SMA segments isolated from male and female gerbils using laser-scanning microscopy and microfluorometry. The gender difference of the LNNA-induced tone was compared, in the same vessel segments, to tone induced by 150 mM K^+^ and endothelin-1, neither of which showed an apparent gender-difference. Interestingly, LNNA-induced tone in male SMAs was observed in protocols that included changes in intramural pressure, but not when the intramural pressure was held constant. LNNA in male SMAs did not increase the global Ca^2+^ concentration in smooth muscle cells but increased the Ca^2+^ sensitivity. This increase in the Ca^2+^ sensitivity was abolished in the presence of the guanylyl cyclase inhibitor ODQ or by extrinsic application of either the nitric oxide (NO)-donor DEA-NONOate or the cGMP analog 8-pCPT-cGMP. The rho-kinase blocker Y27632 decreased the basal Ca^2+^ sensitivity and abolished the LNNA-induced increase in Ca^2+^ sensitivity in male SMAs. Neither LNNA nor Y27632 changed the Ca^2+^ sensitivity in female SMAs. The data suggest that the gender difference in LNNA-induced tone is based on a gender difference in the regulation of rho-kinase mediated Ca^2+^ sensitivity. Rho-kinase and NO thus emerge as critical factors in the regulation of cochlear blood flow. The larger role of NO-dependent mechanisms in male SMAs predicts greater restrictions on cochlear blood flow under conditions of impaired endothelial cell function.

## Introduction

Regulation of blood flow is essential for cochlear function and hearing. Both, neuronal activity and the generation of the endocochlear potential, which are essential for hearing, are exquisitely sensitive to ischemia and oxidative stress [1]–[4]. Consistent with the requirement to control cochlear blood flow within narrow limits, evidence for flow regulation has been observed *in vivo* as well as *in vitro* [5]–[7].

The main blood supply to the cochlea originates from the anterior inferior cerebellar artery that feeds the spiral modiolar artery (SMA) which is located in the center (modiolus) of the cochlea. The SMA supplies the capillary beds in the modiolus, where the cochlear innervation is housed, and branches into radiating arterioles that supply the capillary beds in the cochlear lateral wall, where the endocochlear potential is generated.

Increases in the intramural pressure cause constriction of the SMA. This myogenic response is enhanced by inhibition of nitric oxide synthase (NOS) with L-N^G^-Nitro-Arginine (LNNA). Interestingly, male SMAs exhibit a significantly greater myogenic response in the presence of the NOS inhibitor than female SMAs [7]. This finding raises the question, whether female SMAs are simply weaker and therefore display smaller constrictions, whether LNNA causes in male SMAs a larger increase in the cytosolic Ca^2+^ concentration, or a greater increase in the Ca^2+^ sensitivity of the contractile myofilaments.

In general, myogenic responses depend on a pressure-induced Ca^2+^ influx mainly via L-type Ca^2+^ channels. This Ca^2+^ influx leads to an increase in the cytosolic Ca^2+^ concentration [8] and causes a vasoconstriction dependent on the Ca^2+^ sensitivity of the myofilaments [9]. The Ca^2+^ sensitivity is determined by the relative activity of myosin light chain kinase and myosin light chain phosphatase. Myosin light chain phosphatase activity itself is heavily regulated [10], [11]. Phosphorylation of myosin light chain by myosin light chain kinase leads to smooth muscle constriction, whereas dephosphorylation by myosin light chain phosphatase causes smooth muscle relaxation. Rho-A and the associated rho-kinase are one of the most important mechanisms to increase Ca^2+^ sensitivity. Rho-kinase phosphorylates myosin phosphatase target subunit 1 and thereby inhibits myosin light chain phosphatase [12].

Prior studies have demonstrated that the SMA is endowed with L-type voltage-dependent Ca^2+^ channels that control Ca^2+^ influx and vasoconstriction [13] and further, the SMA has been shown to express rho-kinase that controls Ca^2+^ sensitivity of the myofilaments [14]. These findings support the hypothesis that the gender difference in the LNNA-induced tone is based on a gender difference in the magnitude of the LNNA-induced increase in the cytosolic Ca^2+^ concentration or a difference in the Ca^2+^ sensitivity.

In the present study, we isolated, pressurized and superfused SMAs from male and female gerbils. Vascular diameter and changes in the global cytosolic Ca^2+^ concentration were monitored simultaneously by laser scanning microscopy and microfluorometry using the Ca^2+^ indicator fluo4. The results reveal that male and female SMAs do not differ in their maximal contractile strength and that the greater LNNA-induced tone in male SMAs is due to a greater Ca^2+^ sensitivity that depends on rho-kinase and cGMP. From this study, NO and rho-kinase emerge as critical factors in the regulation of cochlear blood flow. The larger role of NOS-dependent mechanisms in the male SMA predicts greater restrictions on cochlear blood flow under conditions of impaired endothelial cell function.

## Materials and Methods

### Ethics Statement

All procedures involving animals were approved by the Institutional Animal Care and Use Committee at Kansas State University (IACUC#: 2613 and 2961). Male and female gerbils, 5–11 weeks of age (Charles River, Wilmington, MA), were deeply anesthetized with tri-bromo-ethanol (560 mg/kg i.p) and sacrificed by decapitation. Temporal bones were removed and spiral modiolar arteries (SMAs) were isolated by microdissection at 4°C.

### Pressurization and superfusion

Methods for the pressurization and superfusion have been described previously [7], [15]. Briefly, segments of the SMA were transferred into a warm (37°C) custom-built bath chamber that holds an approximate volume of 70 µl and that was mounted on the stage of an inverted microscope (Axiovert 200, Carl Zeiss, Göttingen, Germany). One end of the vessel segment was cannulated using a motorized system of concentric pipettes (Wangemann Instruments, Kansas State University) and the other end was closed with an occluder. Pipettes were prepared on a custom-built micro-forge (Wangemann Instruments). A hydrostatic pressure column was used to apply pressure via the pipette system. Pressurized vessels were maintained at 37°C and superfused at a rate of 1 ml min^−1^. The temperature control entailed a triple system that maintained the superfusate, the bath chamber and the 40x oil-immersion microscope objective at 37°C (Warner instruments, Hamden, CT).

### Solutions and drugs

PSS solution contained (in mM) 150 NaCl, 5 HEPES, 3.6 KCl, 1 MgCl_2_, 1 CaCl_2_ and 5 glucose, pH 7.4. Ca^2+^-free solution contained (in mM) 150 NaCl, 5 HEPES, 3.6 KCl, 1 MgCl_2_, 1 EGTA and 5 glucose, pH 7.4 and 150 mM K^+^ solution contained (in mM) 150 KCl, 5 HEPES, 1 MgCl_2_, 1 CaCl_2_ and 5 glucose, pH 7.4. Compounds added to these solutions included 10 µM L-N^G^-nitro-arginine (LNNA, Sigma-Aldrich, St. Louis, USA), 10 nM endothelin-1 (ET-1, Sigma-Aldrich), 100 µM papaverine (Sigma-Aldrich), 8 µM diethylammonium (Z)-1-(N,N-diethylamino)diazen-1-ium-1,2-diolate (DEA-NONOate, Cayman Chemical Company, Ann Arbor, Michigan, USA), 10 µM trans-4-[(1R)-1-aminoethyl]-N-4-pyridinyl-cyclo-hexane-carboxamide dihydrochloride (Y27632, Cayman), 10 µM 1H-[1], [2], [4] oxadiazolo [4,3-a] quinoxalin-1-one (ODQ, Cayman), 100 µM 8-bromo-guanosine 3′,5′-cyclic monophosphate sodium salt (8-Br-cGMP, Sigma-Aldrich) and 100 µM or 500 µM 8-(4-chlorophenylthio)-guanosine 3′,5′-cyclic monophosphate sodium salt (8-pCPT-cGMP, Sigma-Aldrich). In some experiments, the Ca^2+^ concentration was increased to 3 and 10 mM by addition of CaCl_2_. Stock solutions of 20 mM LNNA, 10 mM Y27632, 50 mM ODQ and 250 mM papaverine were prepared in DMSO. The final concentration of DMSO did not exceed 0.1%. A stock solution of 2 µM endothelin-1 was prepared in PSS, stock solutions of 1.5 mM 8-Br-cGMP and 1.5 mM 8-pCPT-cGMP were prepared in deionized H_2_O, and a stock solution of 8 mM DEA-NONOate was prepared in 0.01 N NaOH. Addition of the DEA-NONOate stock to the final solution did not change the pH by >0.01 pH-units.

### Measurements of vascular diameter and myogenic tone

Brightfield images of pressurized vessel segments were obtained by laser scanning microscopy at a rate of 1 image/s (LSM 510 Meta, Carl Zeiss). Images were analyzed using a custom program written in IDL 6.3 (RSI, now Exelis, Boulder, CO, USA) by Withrow Gil Wier (University of Maryland, MD, USA) and analyzed with a custom program written in Origin 6.0 (Microcal, now OriginLab, Northampton, MA, USA) by P. Wangemann (Kansas State University, KS, USA). Myogenic tone (%) was calculated as (ID_passive_ – ID_active_) ×100/ ID_passive_, where ID_passive_ is the inner diameter in Ca^2+^-free solution and ID_active_ is the inner diameter in PSS or 150 mM K^+^ solution with or without compounds.

### Measurements of fluorescence intensity and global cytosolic Ca^2+^

Changes in the global Ca^2+^ concentration of the smooth muscle cells were monitored by measurements of fluorescence intensity using the indicator dye fluo4. Dyes loaded virtually exclusively into the smooth muscle cells so that changes in the fluorescence intensity could be attributed to the cytosol of smooth muscle cells. Fluorescence images of the pressurized vessel segments were obtained simultaneously with brightfield images. Pressurized vessel segments were loaded by 15 min incubation in 2.5 µM fluo4-AM (Invitrogen, Carlsbad, CA, USA) at 37°C. Fluo4 was excited by a 488 nm argon laser and emissions were filtered by a 488 notch, a 490 nm dichroic and a 505 nm long pass filter. Global Ca^2+^ concentration changes were obtained by averaging fluo4 intensity measurements from 5 smooth muscle cells per vessel segment. Data presented are averages from multiple vessel segments.

### Measurements of Ca^2+^ sensitivity

Vascular diameter and changes in global Ca^2+^ were simultaneously measured in vessel segments pressurized at 60 cmH_2_O. Following equilibration for 15 min in PSS, arteries were superfused with PSS solutions containing 0, 1, 3 and 10 mM Ca^2+^ each for 2 min. Data points were obtained by averaging diameter and fluorescence intensity measurements over the last 30 s of each Ca^2+^ step and normalizing these values against the average value obtained in PSS containing 1 mM Ca^2+^. Data points from individual vessels were fitted to a modified Hill equation:
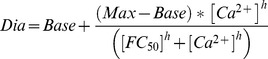
where *Dia* is the normalized diameter, *Base* is the minimal diameter independently obtained in the presence of 10 nM ET-1 to be 53% in female and 55% in male SMA, *Max* is the diameter in PSS containing 1 mM Ca^2+^, *[Ca^2+^]* is the normalized cytosolic Ca^2+^ concentration, *h* is the slope coefficient, and *FC_50_* is the fold-change in the global cytosolic Ca^2+^ concentration that is necessary for a half-maximal constriction. The slope coefficient *h* was arbitrarily set to −5.2 and *Max* was clamped to 100%. Two *FC_50_* values describing a control and an experimental period were obtained from each vessel segment. For presentation, data points were averaged and curves representing the averaged *FC_50_* values were drawn.

For evaluating the effect of the NO donor on Ca^2+^ sensitivity in the presence of 10 µM LNNA, a stock solution (8 mM) of the NO donor DEA-NONOate was prepared in 0.01 N NaOH where the NO donor is stable. Prior to the beginning of every 2 min step in the protocol, 10 µl of the stock was added to 10 ml of the superfusing solution (PSS containing 10 µM LNNA and 0, 1, 3 or 10 mM Ca^2+^ at pH = 7.4) and quickly mixed (within 15 seconds) to obtain a concentration of 8 µM DEA-NONOate. With a reported decomposition rate of 1.5 mol NO per mol of DEA-NONOate and the half-life of 2 min at pH = 7.4 and 37°C, the concentration of the released NO at the end of the 2 min step in the protocol is expected to drop to ∼6 µM, a saturating concentration that is approximately 30-fold higher than the *EC_50_*_­_ reported for rat aorta [16].

### Statistics

The number of experiments (n) represents the number of vessel segments. Data are presented as mean ± sem. Each mean consists of vessel segments that originated from more than one animal. Statistical significance for differences in tone between multiple treatments was obtained on raw data by 2-way repeated-measures ANOVA (Sigma-Stat 3.0, Systat, San Jose, CA, USA). Statistical significance for *FC_50_* values was obtained by paired Student's *t*-test. Significance was assumed at p<0.05.

## Results

### NOS inhibition enhances tone in response to changes in intramural pressure

**Figure 1:**
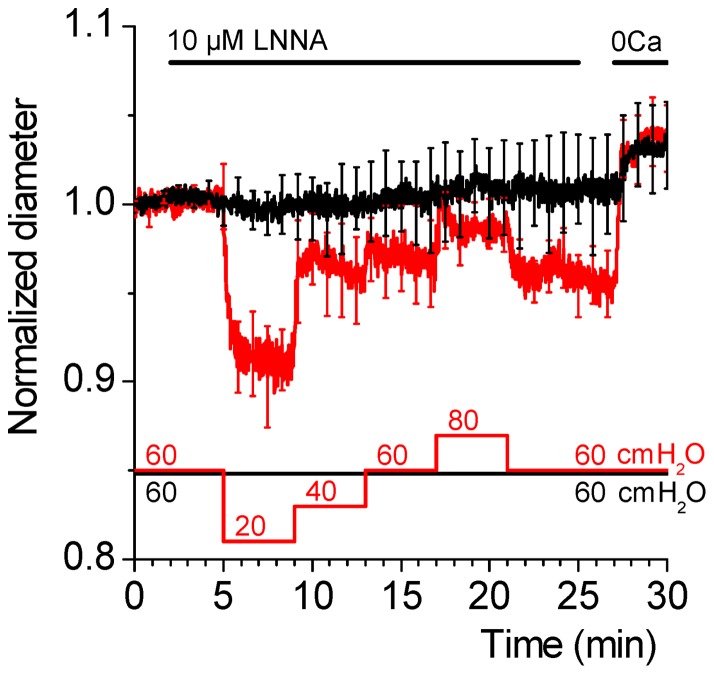
NOS inhibition with LNNA enhances tone in response to intramural pressure changes but not in the presence of a constant pressure. Vascular diameter was monitored in pressurized segments of male SMAs that were superfused with PSS solution with and without 10 µM LNNA and with Ca^2+^ free solution (0Ca), as indicated by *bars* located above the traces. During the period of superfusion with LNNA, vessel segments were subjected either to a constant intramural pressure of 60 cmH_2_O (*black trace*) or different intramural pressures as indicated (*red trace*). Intramural pressures are indicated by *bars* located below the traces. Note that the vascular diameter at 60 cmH_2_O decreased in vessel segments that were exposed to pressure variations but not in vessel segments that were exposed to a constant pressure and that the diameter in Ca^2+^ free solution was similar in both groups. Traces represent averages of 5 vessel segments. Data were acquired at 1 s intervals, however, error bars (sem) were plotted only every 50 s.

Nitric oxide (NO) opposes myogenic constrictions in most large and small arteries and thereby exerts a vasoprotective role in response to physiological stimuli such as pressure-induced stretch and flow-induced shear that cause vasoconstriction [17], [18]. In the SMA, NO-dependent vasoregulation is gender-dependent, since NOS inhibition increased myogenic tone in male, but not the female SMAs [7]. To better understand the nature of the LNNA-induced vascular tone in the SMA, we determined whether LNNA-induced vascular tone develops at a constant pressure or requires pressure changes. SMA from male gerbils were pressurized at 60 cmH_2_O and superfused with 10 µM LNNA, during which the pressure was either held constant at 60 cmH_2_O to provide constant stretch or altered between 20 and 80 cmH_2_O (Fig. 1). When pressure changes were introduced during the 23 min period of incubation with LNNA, myogenic tone was observed as previously reported (Fig. 1, *red trace*). Interestingly, tone was not enhanced, when the pressure was held constant (Fig. 1, *black trace*). These results demonstrate that NOS inhibition in conjunction with changes in the intramural pressure promotes myogenic vasoconstriction in the male SMA.

### Male and female SMA do not differ in their maximal contractility

**Figure 2:**
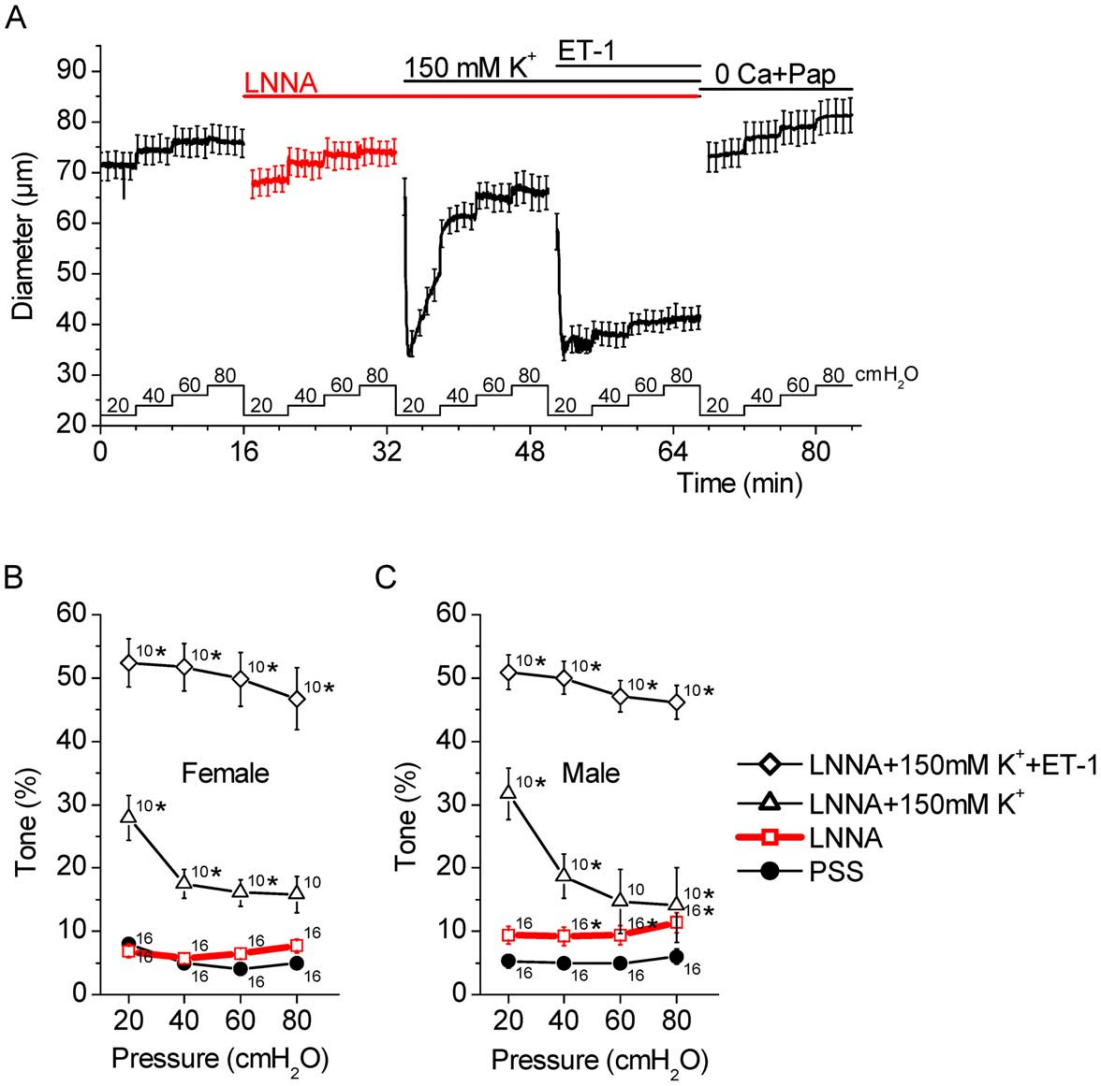
LNNA-induced tone is gender-dependent but K^+^ and ET-1 induced tone is gender-independent. Vascular diameter was monitored at intramural pressures of 20, 40, 60 and 80 cmH_2_O while vessel segments were superfused with PSS solution to which 10 µM LNNA was added, with 150 mM K^+^ solution to which 10 µM LNNA and 10 nM ET-1 were added, and with Ca^2+^ free solution to which 100 µM papaverine was added (0 Ca+Pap). A) Summary of diameter measurements performed in male SMAs. The superfusion protocol is indicated by *bars* located above the data trace. Intramural pressures are indicated by *bars* located below the data trace. The data trace represents the average of 10 vessel segments. Data were acquired at 1 s intervals, however, for clarity, error bars (sem) are plotted only every 50 s. B) Summary of the calculated tone in female SMAs. C) Summary of the calculated tone in male SMAs. The legend provided in C applies to B and C. Numbers next to symbols in B and C represent the number of vessel segments. Stars indicate a significant change in tone at a given pressure compared to the prior condition.

In contrast to male SMAs, LNNA did not enhance myogenic tone in female SMAs [7]. It could be argued that intrinsic differences in the contractility of male and female SMAs underlie this gender difference. Thus, we compared the effects of LNNA and two vasoconstrictors, K^+^ and ET-1, in male and females SMAs. Tone was measured in PSS, 10 µM LNNA, 150 mM K^+^ and 10 nM ET-1 (Fig. 2). The rationale for applying 150 mM K^+^ was to induce complete membrane depolarization and the rationale for applying 10 nM ET-1 was to induce a maximal tone. Consistent with previous results, addition of LNNA had no effect on the myogenic tone in female SMAs (Fig. 2B) but increased myogenic tone in male SMAs (Fig. 2C). Myogenic tone was increased in male and female SMA by 150 mM K^+^ and further increased by 10 nM ET-1. In contrast with the gender difference in LNNA-induced myogenic tone, there was no difference in the myogenic tone in the presence of PSS, 150 mM K^+^ or ET-1. These results demonstrate that the lack of LNNA-induced tone in female SMA is not due to a lesser contractility.

### LNNA does not increase tone in male SMA via an increase in global Ca^2+^

**Figure 3:**
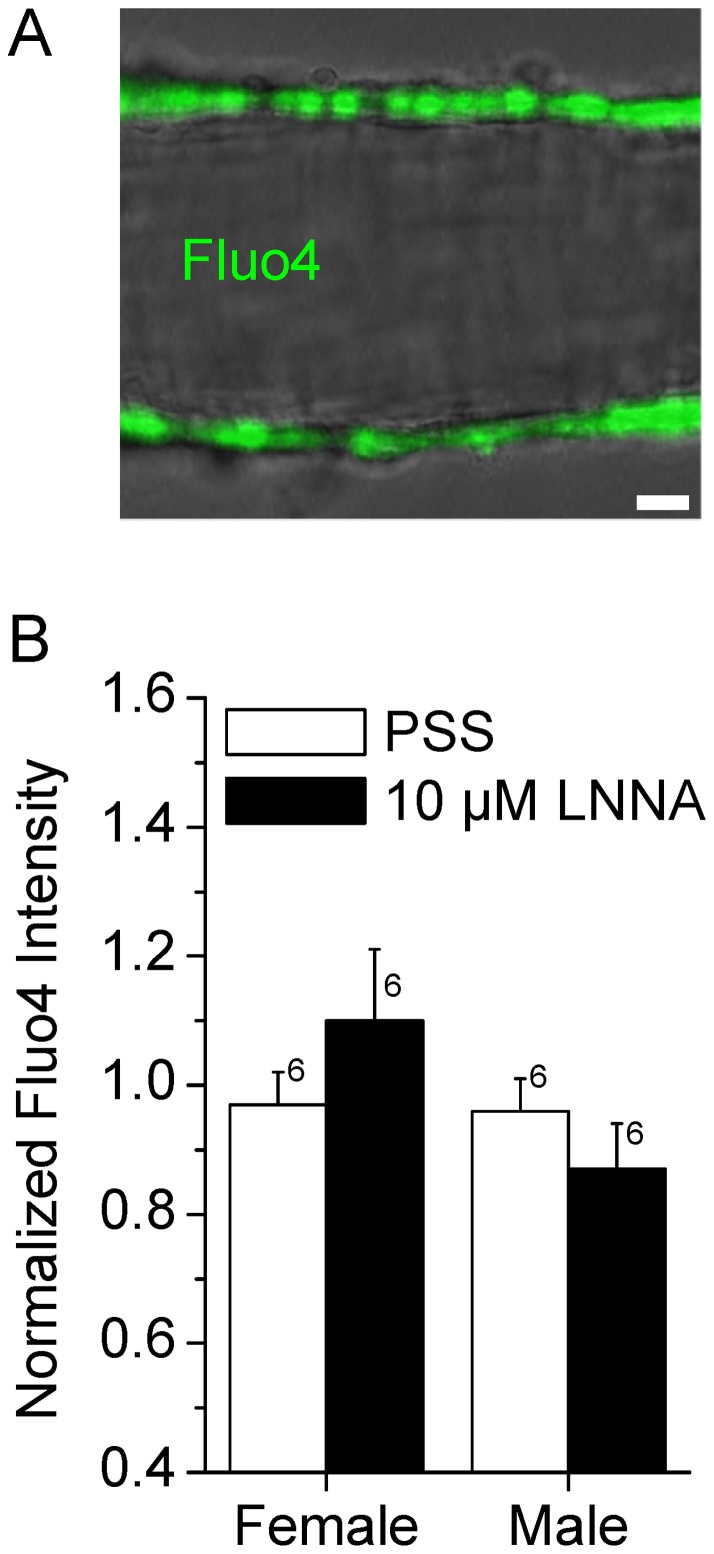
NOS inhibition with LNNA does not increase the cytosolic Ca^2+^ concentration in smooth muscle cells of the SMA. Male and female SMAs were pressurized at 60 cmH_2_O and superfused with PSS to which 10 µM LNNA was added. Changes in the global cytosolic Ca^2+^ concentration were measured as fluo4 fluorescence intensity. A) Superimposed image of laser-scanning bright-field image (*grey scale*) and a fluorescence image of a pressurized SMA loaded with fluo4 (*green*). Note that fluorescence was emitted solely from smooth muscle cells. Scale bar  = 10 µm. B) Changes in global cytosolic Ca^2+^ concentration. Numbers next to the error bars represent the number of SMA segments. No significant differences were observed.

NOS inhibition may increase myogenic tone via multiple mechanisms including increase in cytosolic Ca^2+^ concentration. Changes in the cytosolic Ca^2+^ concentration in smooth muscle cells of the SMA were monitored using the fluorescent indicator dye fluo4. The global cytosolic Ca^2+^ concentration in smooth muscle cells of the SMA remained unchanged by 10 µM LNNA. Similar observations were made in both genders (Fig. 3B). Control experiments revealed that time had no effect on the fluorescence intensities (0.9±0.1 vs 0.9±0.1, n = 7). The observation demonstrates that LNNA does not increase tone via an increase in the global cytosolic Ca^2+^ concentration.

### LNNA increases Ca^2+^ sensitivity in male but not female SMA

**Figure 4:**
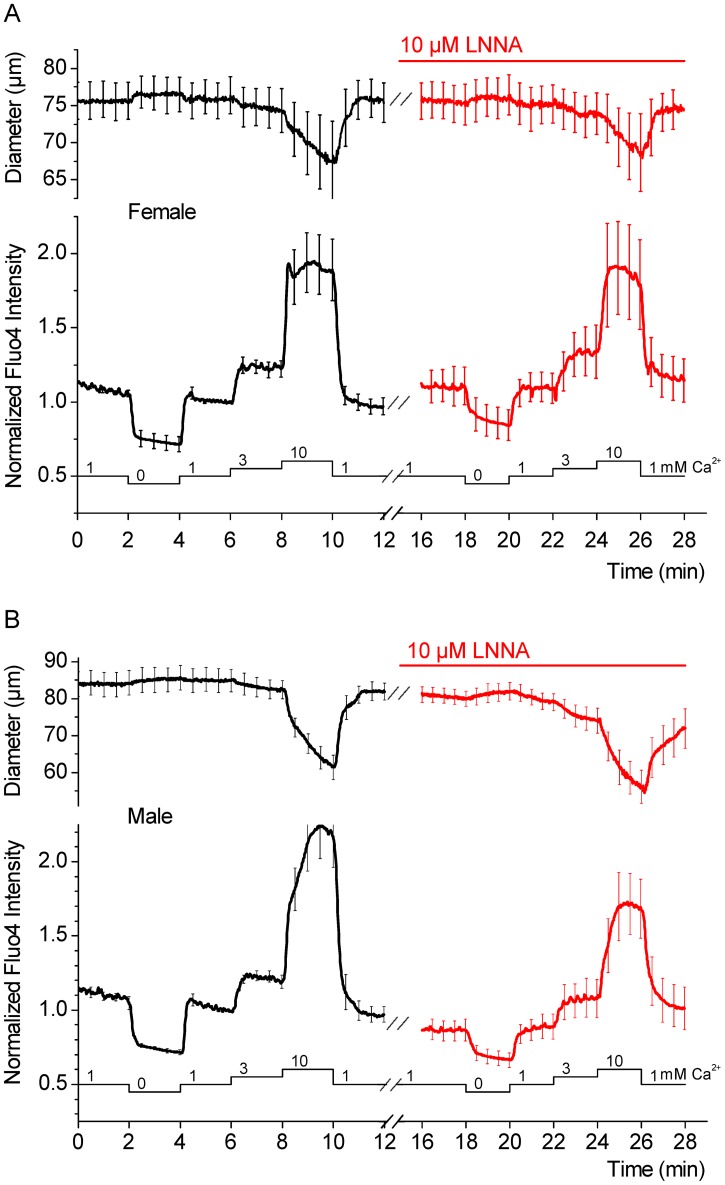
Protocol for measuring the Ca^2+^ sensitivity in the SMA. Changes in the vascular diameter and in the fluorescence intensity of the indicator dye fluo4 were measured simultaneously. Vessel segments were pressurized at 60 cmH_2_O and superfused with PSS with and without 10 µM LNNA as indicated by the *bar* above the data traces. The Ca^2+^ concentration in the superfusate was varied between 0, 1, 3 and 10 mM, as indicated by the *bar* below the data traces. A) Summary of diameter measurements and corresponding changes in global cytosolic Ca^2+^ concentration reflected by fluo4 fluorescence intensity obtained in female SMAs. B) Summary of diameter measurements and corresponding changes in global cytosolic Ca^2+^ concentration reflected by fluo4 fluorescence intensity obtained in male SMAs. Traces represent averages of 6 vessel segments. Data were acquired at 1 s intervals, however, for clarity, error bars (sem) are plotted only every 50 s.

**Figure 5:**
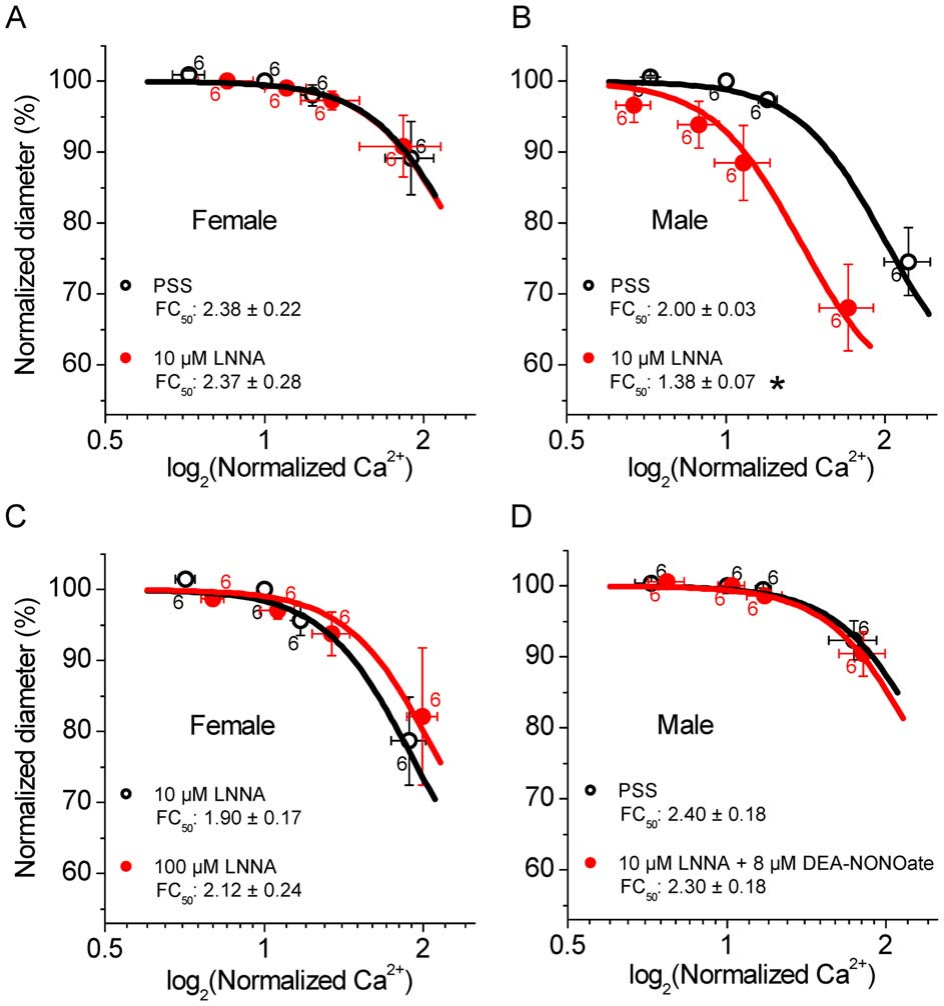
LNNA increases the Ca^2+^ sensitivity in male but not female SMAs and DEA-NONOate reverses the LNNA effect in male SMAs. A) Ca^2+^ sensitivity in female SMAs in PSS and 10 µM LNNA. B) Ca^2+^ sensitivity in male SMAs in PSS and 10 µM LNNA. C) Ca^2+^ sensitivity in female SMAs in 10 µM and 100 µM LNNA. D) Ca^2+^ sensitivity in male SMAs in PSS and in 10 µM LNNA + 8 µM DEA-NONOate. Numbers next to symbols represent the number of SMA segments and error bars represent sem. Average *FC_50_* ± sem are given and significant differences in the *FC_50_* values are marked (*star*).

**Figure 6:**
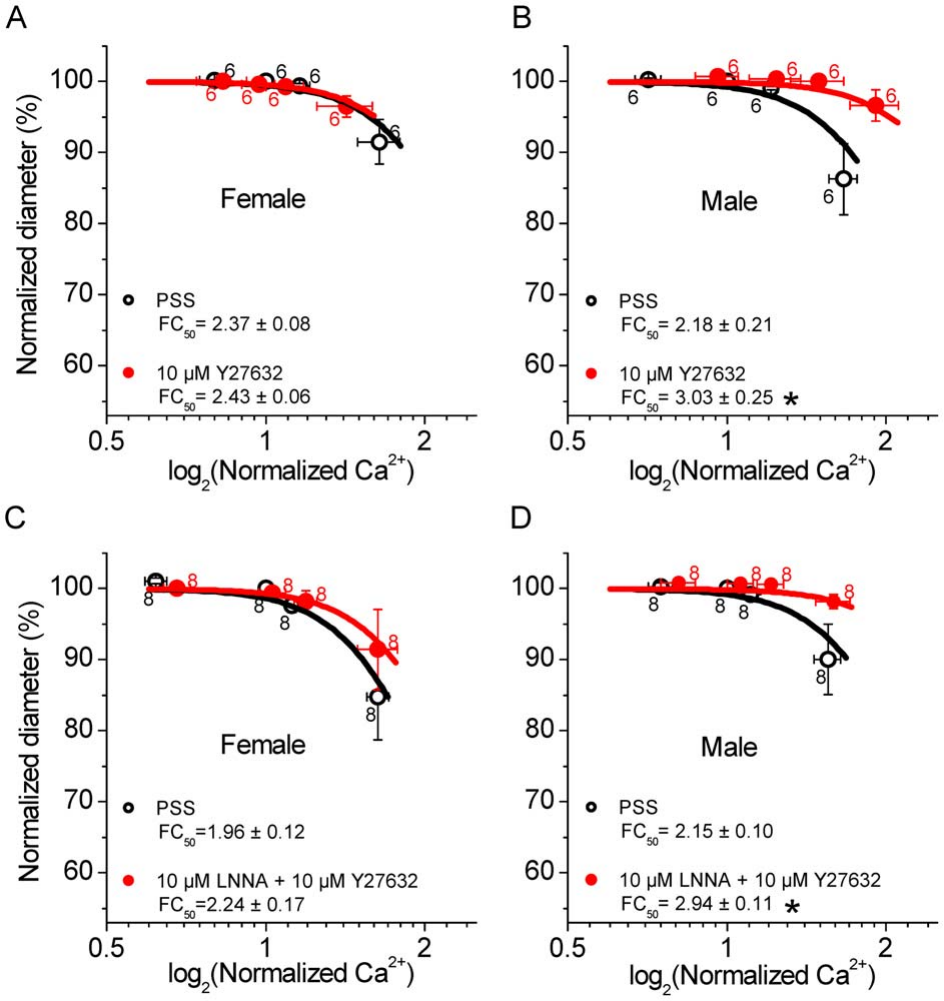
Y27632 lowers the Ca^2+^ sensitivity in male but not female SMAs and abolishes the LNNA effect in male SMAs. A) Ca^2+^ sensitivity in female SMAs in PSS and 10 µM Y27632. B) Ca^2+^ sensitivity in male SMAs in PSS and 10 µM Y27632. C) Ca^2+^ sensitivity in female SMAs in PSS and in 10 µM LNNA + 10 µM Y27632. D) Ca^2+^ sensitivity in male SMAs in PSS and in 10 µM LNNA +10 µM Y27632. Numbers next to symbols represent the number of vessel segments and error bars represent sem. Average *FC_50_* ± sem values are given and significant changes in the *FC_50_* values are marked (*star*).

**Figure 7:**
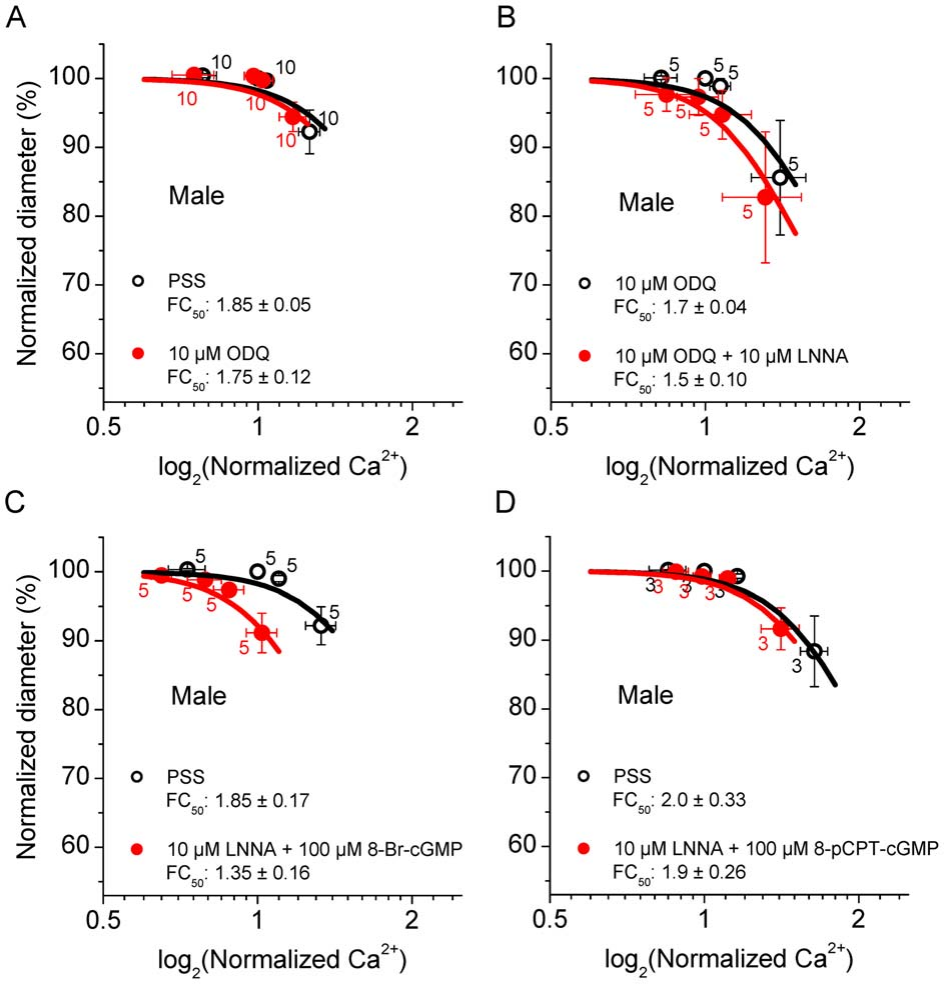
ODQ does not affect the basal Ca^2+^ sensitivity but ODQ and 8-pCPT-cGMP greatly diminish the LNNA-induced increase in the Ca^2+^ sensitivity in male SMAs. A) Ca^2+^ sensitivity in male SMAs in PSS and 10 µM ODQ. B) Ca^2+^ sensitivity in male SMAs in 10 µM ODQ and 10 µM ODQ + 10 µM LNNA. C) Ca^2+^ sensitivity in male SMAs in PSS and 10 µM LNNA + 100 µM 8-Br-cGMP. D) Ca^2+^ sensitivity in male SMAs in PSS and 10 µM LNNA + 100 µM 8-pCPT-cGMP. Numbers next to symbols represent the number of vessel segments and error bars represent sem. Average *FC_50_* ± sem values are given.

NO regulates vascular tone in some vessels through a decrease of the Ca^2+^ sensitivity [19]. Such a mechanism would be consistent with the observed lack of an LNNA-induced increase in the smooth muscle global Ca^2+^ concentration. To demonstrate whether LNNA alters the Ca^2+^ sensitivity, vascular diameter and the global cytosolic Ca^2+^ concentration in the smooth muscle cells were monitored simultaneously in male and female SMA (Fig. 4). Alterations in the global cytosolic Ca^2+^ concentration were induced by altering the Ca^2+^ concentration in the superfusate to 0 (with 1 mM EGTA), 1, 3 or 10 mM Ca^2+^. Changes in the diameter were plotted against changes in the global cytosolic Ca^2+^ concentration (Figs. 5, 6, 7). Ca^2+^ sensitivity was quantified as a fold-change in the global cytosolic Ca^2+^ concentration that is necessary to achieve a half-maximal constriction (*FC_50_*). NOS inhibition with 10 µM LNNA produced no significant effect on the Ca^2+^ sensitivity in female SMA, however, caused a marked increase in the Ca^2+^ sensitivity in male SMA (Fig. 5A and B). These observations suggest that the gender difference of LNNA-induced tone is a consequence of the gender difference in the regulation of the Ca^2+^ sensitivity.

It could be argued, that the lack of an LNNA-induced increase in the Ca^2+^ sensitivity in female SMA is the result of an insufficient inhibition by 10 µM LNNA. However, no significant difference was found between the Ca^2+^ sensitivity in the presence of 10 and 100 µM LNNA (Fig. 5C). This observation dismisses the possibility of an insufficient NOS inhibition in female SMAs.

The effect of LNNA in male SMAs implies that the Ca^2+^ sensitivity is lowered by NO. Consistent with this interpretation is the finding that the NO-donor, 8 µM DEA-NONOate completely abolished the effect of 10 µM LNNA in male SMA (Fig. 5D).

### Inhibition of rho-kinase reduces Ca^2+^ sensitivity in male but not female SMA

Y27632-sensitive rho-kinase is one of the most common regulators of the Ca^2+^ sensitivity in vascular smooth muscle cells [20]. The observed gender difference in the regulation of Ca^2+^ sensitivity by NO could thus be based on a difference in rho-kinase activity. Inhibition of rho-kinase with 10 µM Y27632 caused no change in the Ca^2+^ sensitivity in female SMA, however, caused a decrease in the Ca^2+^ sensitivity in male SMAs (Fig. 6A and 6B). Further, 10 µM Y27632 had no effect on the Ca^2+^ sensitivity in the presence of 10 µM LNNA in female SMA, however, Y27632 abolished the LNNA-induced increase in the Ca^2+^ sensitivity in male SMA (Fig. 6C and 6D). This observation suggests that the gender difference of LNNA-induced myogenic tone is a consequence of the gender difference in the basal rho-kinase activity and that NO regulates the Ca^2+^ sensitivity in the male SMA via the regulation of rho-kinase.

### Inhibition of guanylyl cyclase or extrinsic supply of cGMP abolishes LNNA-induced increase in Ca^2+^ sensitivity in male SMA

The effects of NO in vascular smooth muscle cells are commonly mediated by the activation of soluble guanylyl cyclase leading to an increase of the second messenger cGMP [21]. cGMP targets several mechanisms including potentiation of K^+^ channel activity [22], inhibition of Ca^2+^ channels [23] and regulation of contraction and rho-kinase [24]. The observed LNNA-induced increase in the Ca^2+^ sensitivity in the male SMA could thus be due to loss of activation of guanylyl cyclase and decreased formation of cGMP. Interestingly, application of the soluble guanylyl cyclase inhibitor 10 µM ODQ did not change the basal Ca^2+^ sensitivity of the male SMA (Fig. 7A). However, in the presence of 10 µM ODQ, the LNNA-induced increase in the Ca^2+^ sensitivity in male SMA was greatly diminished (Fig. 7B), suggesting a cGMP-dependent mechanism. Application of the cGMP analog, 100 µM 8-Br-cGMP failed to reverse the LNNA-induced increase in the Ca^2+^ sensitivity (Fig. 7C). However, the same concentration of 100 µM 8-pCPT-cGMP, a more potent cGMP analog [25] with greater membrane permeability and higher resistance to phosphodiesterase activity, completely abolished the LNNA-induced increase in the Ca^2+^ sensitivity (Fig. 7D). A similar outcome was obtained with a higher concentration of 500 µM 8-pCPT-cGMP (*FC_50_* in PSS: 2.1±0.2 and *FC_50_* in 500 µM 8-pCPT-cGMP: 2.0±0.2; n = 3). These observations are consistent with a mechanism whereby NO stimulates guanylyl cyclase to generate cGMP and cGMP leads to inhibition of rho-kinase. The lack of an effect of ODQ itself suggests the presence of compensatory mechanisms that regulate rho-kinase and/or the Ca^2+^ sensitivity in the absence of cGMP.

## Discussion

The most salient findings of the present study are that changes in intramural pressure during NOS inhibition by LNNA enhances myogenic vasoconstrictions in the male but not in the female SMA, that this gender difference is neither a manifestation of a difference in the maximal achievable contractility, nor the consequence of an increase in global Ca^2+^ concentration, but the result of a difference in the rho-kinase mediated increase in Ca^2+^ sensitivity. The results suggest that NO controls the Ca^2+^ sensitivity of the male SMA via the regulation of rho-kinase in a cGMP-dependent manner.

Myogenic tone in male SMAs was enhanced during NOS inhibition when the vessel was subjected to a protocol of pressure changes but not when subjected to a single constant intramural pressure (Fig. 1). This observation suggests that multiple mechanisms are involved in mediating pressure-dependent effects. It is conceivable that transient shear stimuli were induced during the pressure changes and that the combination of pressure and shear stimuli triggered molecular transformations in the endothelial and smooth muscle cells of the SMA that were absent under conditions of constant pressure. Both stretch and shear stimuli can lead to an upregulation of endothelial NOS activity [17], [18]. Our results suggest that NO plays a role in the male SMA in opposing myogenic constrictions induced by shear and/or pressure changes.

In the coronary and cerebral vasculature, it is well-established that the NO-induced reduction in vascular tone is gender-dependent [26]. The consensus emerging out of several studies is that NO plays a major role in mediating vasodilation and suppressing myogenic tone in females [26]–[28]. Basal NO levels are elevated by estrogen, which underlies the greater sensitivity of females to endothelium-dependent vasodilation [28]–[30]. NO masks the myogenic response in female cerebral vessels *in vitro* and large arterioles *in vivo* [27], [28], [31]. Indeed, NOS inhibition is frequently used to uncover myogenic constriction in females that is comparable in magnitude to or even greater than in males [27], [28]. In contrast, NOS inhibition in the SMA uncovered a significant myogenic tone in males, but not in the females (Fig. 2B vs 2C). This finding suggests that NO may play a greater role in the regulation of cochlear blood flow in males than in females.

NO can decrease the cytosolic Ca^2+^ concentration of smooth muscle cells by different mechanisms. Some of these mechanisms involve activation of K^+^ channels, hyperpolarization of the membrane potential and closure of voltage-dependent, mainly L-type, Ca^2+^ channels that mediate Ca^2+^ influx from the extracellular medium. For instance, NO hyperpolarizes the membrane by activation of large-conductance Ca^2+^ activated K^+^ channels either directly [32] or via phosphorylation [22], [33], by acting on K_ATP_ K^+^ channels [34], or by causing the release of an endothelium-derived hyperpolarizing factor that acts on the smooth muscle cells [35]. Another mechanism by which NO hyperpolarizes the membrane is through an increase in the frequency of Ca^2+^ sparks, which are spatially confined Ca^2+^ releases from the sarcoplasmic reticulum that occur via ryanodine-sensitive Ca^2+^ channels and activate closely apposed large-conductance Ca^2+^ activated K^+^ channels [36]. Other mechanisms by which NO decreases the cytosolic Ca^2+^ concentration of smooth muscle cells entails stimulation of the Ca^2+^ ATPase in the sarcoplasmic reticulum that increases the uptake of Ca^2+^ into the sarcoplasmic reticulum [37]. All of these actions limit the cytosolic Ca^2+^ concentration and cause relaxation of smooth muscle cells. Common to all of these mechanisms is that NOS inhibition with LNNA would be expected to lead to a vasoconstriction via an increase in the cytosolic Ca^2+^ concentration. Interestingly, LNNA-mediated NOS inhibition in the male SMA increased tone without an increase in the global cytosolic Ca^2+^ concentration (Fig. 3B). This observation establishes the SMA as an arteriole that is different from other commonly studied arterioles.

NO not only modulates the cytosolic Ca^2+^ concentration in smooth muscle cells but also the Ca^2+^ sensitivity of the myofilaments. NO has been shown to inhibit rho-kinase activity leading to disinhibition of myosin light chain phosphatase, since rho-kinase increases the Ca^2+^ sensitivity by inhibiting myosin light chain phosphatase [20], [38]. The observations that NOS inhibition caused an increase in the Ca^2+^ sensitivity (Fig. 5B) and that this increase was abolished by an extrinsic supply of NO (Fig. 5D) demonstrates that NO-mediated modulation of the Ca^2+^ sensitivity is a major pathway of controlling tone in the male SMA. The finding that the LNNA-mediated increase in the Ca^2+^ sensitivity was abolished by Y27632 (Fig. 6D) establishes rho-kinase as the main determinant of the Ca^2+^ sensitivity and the target of regulation by NO in the male SMA.

A striking gender difference was found between male and female SMAs in the action of NO. LNNA-mediated NOS inhibition increased tone and the Ca^2+^ sensitivity in males, but not in females (Fig. 2B vs 2C and Fig. 5A vs 5B). The possibility that NOS inhibition was incomplete in females due to a higher rate of NO production was ruled out by the observation that a 10-fold higher concentration of LNNA failed to increase the Ca^2+^ sensitivity (Fig. 5C). The lack of an effect of LNNA on the Ca^2+^ sensitivity in female SMAs may be attributable to a lower basal rho-kinase activity. This hypothesis is supported by the observation that rho-kinase inhibition did not decrease basal Ca^2+^ sensitivity in female SMAs whereas an inhibitory effect was observed in male SMAs (Fig. 6A vs 6B). A lower basal rho-kinase activity in female SMAs is consistent with the observation that estrogen suppresses rho-kinase expression and function [39], [40]. In this context, it is interesting that no gender difference was observed in the ET-1 induced tone since ET-1 mediated vasoconstriction depends largely on an increase in the rho-kinase mediated Ca^2+^ sensitivity [14] (Fig. 2B vs 2C). Taken together, these observations suggest that basal rho-kinase activity may be lower in females but that, once rho-kinase is activated, there is no apparent gender difference in its ability to maintain a high Ca^2+^ sensitivity.

The most common mechanism through which NO regulates smooth muscle function involves the activation of soluble guanylyl cyclase that leads to increased formation of the second messenger cGMP [24]. cGMP activation of protein kinase G in response to NO elicits the phosphorylation of several proteins that result in the lowering of Ca^2+^ as well as the lowering of Ca^2+^ sensitivity via the inhibition of Rho-A and rho-kinase [24]. Our result that the LNNA-induced increase of the Ca^2+^ sensitivity in the male SMA was profoundly diminished in the presence of the guanylyl cyclase inhibitor ODQ and completely abolished by the cGMP analog is consistent with such a mechanism (Fig. 7). The fact that the inhibition of sGC alone had no effect on the basal Ca^2+^ sensitivity indicates that other mechanisms may be involved. Nevertheless, our results suggest that cGMP is essential for the rho-kinase mediated regulation of Ca^2+^ sensitivity by NO.

In summary, this study demonstrates gender differences in the regulation of rho-kinase mediated Ca^2+^ sensitivity and tone during NOS inhibition and reveals mechanisms by which NOS inhibition induces vasoconstriction in male SMA. These gender differences suggest that loss of NO is likely to have a greater impact on the regulation of cochlear blood flow in males than in females, which is consistent with animal experiments and clinical outcomes that suggest a gender preference in hearing loss, with males being more susceptible [41], [42]. Given the higher propensity of cardiovascular disease in males compared to females [43], our results point to possible mechanisms that may underlie the greater susceptibility of males to age-related hearing loss. Given the promise of rho-kinase inhibitors in treating cardiovascular disorders, the results from this study provide a basis for exploring the use of rho-kinase inhibitors in the treatment of hearing loss in males [44], [45].
